# Dynamical indicators in time series of healthcare expenditures predict mortality risk of older adults following spousal bereavement

**DOI:** 10.1186/s12877-022-02992-x

**Published:** 2022-04-08

**Authors:** Alexandros Katsiferis, Pernille Yde Nielsen, Majken K. Jensen, Rudi G. J. Westendorp

**Affiliations:** 1 Section for Epidemiology, Department of Public Health, University of Copenhagen, Copenhagen, Denmark; 2 Center for Healthy Aging, University of Copenhagen, Copenhagen, Denmark

**Keywords:** Resilience, Bereavement, Complex systems, Health, Aging

## Abstract

**Background:**

The process of aging renders older people susceptible for adverse outcomes upon stress. Various indicators derived from complex systems theory have been proposed for quantifying resilience in living organisms, including humans. We investigated the ability of system-based indicators in capturing the dynamics of resilience in humans who suffer the adversity of spousal bereavement and tested their predictive power in mortality as a finite health transition.

**Methods:**

Using longitudinal register data on weekly healthcare consumption of all Danish citizens over the age of 65 from January 1st, 2011, throughout December 31st, 2016, we performed statistical comparisons of the indicators ‘average’, ‘slope’, ‘mean squared error’, and ‘lag-1 autocorrelation’ one year before and after spousal bereavement, stratified for age and sex. The relation between levels of these indicators before bereavement and mortality hazards thereafter was determined by time to event analysis. We assessed the added value for mortality prediction via the time dependent area (AUC) under the receiver operating characteristic curve.

**Results:**

The study included 934,003 citizens of whom 51,890 experienced spousal bereavement and 2862 died in the first year thereafter. Healthcare consumption is increased, more volatile and accelerating with aging and in men compared to women (all *p*-values < 0.001). All dynamic indicators before bereavement were positively related with mortality hazards thereafter (all *p*-values < 0.001). The average discriminative performance for the 1-year mortality risk of the model with only age as a predictor (AUC: 68.9% and 70.2%) was significantly increased with the addition of dynamical indicators (78.5% and 82.4%) for males and females, respectively.

**Conclusions:**

Dynamic indicators in time series of health care expenditures are strong predictors of mortality risk and could be part of predictive models for prognosis after life stressors, such as bereavement.

**Supplementary Information:**

The online version contains supplementary material available at 10.1186/s12877-022-02992-x.

## Background

Resilience as a term has been used in a variety of different fields to describe a certain system’s ability to preserve specific characteristics or functions when being perturbed by different stressors [1]. Originally developed within the physical sciences, insights from the theory of complex dynamical systems have been extrapolated to understand and predict failure of organisms in the life sciences [2–4]. In human health, resilience to stressors can be interpreted as the potential of a person to bounce back into normal functioning after facing mental or physical disturbances [5–7]. This adaptive capacity of humans diminishes across time due to the process of aging, a phenomenon called frailty, describing the functional decline of various physiological systems rendering older people at an increased vulnerability to stressors [8].

A more formal application of complex dynamical systems theory to human aging has led to the development of indicators for quantifying and estimating resilience [9]. According to theory some systems may exhibit a phenomenon called ‘Critical Slowing Down’ (CSD) during which the system recovers in a slower rate from disturbances [9–12]. This decline of resilience can be manifested as the system approaches a tipping point transitioning from a stable steady state, e.g. health towards a contrasting one, e.g. disease [11, 13]. Using statistical terms, slower recovery rate can be reflected in high temporal autocorrelation, which measures the similarity of system’s dynamics in consecutive intervals (lags), accompanied with high variability, hence fluctuations, from the initial steady state. The aforestated statistical metrics can be found in the literature as Dynamical Indicators of Resilience (DIORs), since they are used to observe subtle changes in the dynamics of a given continuous (physiological) variable [9]. In general, the list of available, suggested DIORs is not restrictive, but ranges from plain univariate indicators such as autocorrelation, standard deviation, skewness and kurtosis [11, 14–16] to multivariate ones such as cross-correlation [17]. Empirical testing of such dynamical indicators in time-series of self-rated health, postural balance, heart rate and physical activity data has provided evidence for their usefulness in the gerontology field, by their ability to quantify physical resilience of older adults and predict their respective recovery potential from stressors in the future [6, 18–22]. The present study will empirically test the epidemiological potential of dynamical indicators in time-series of individuals’ healthcare expenditures available from the Danish (nationwide) registers, with the latter reflecting a person’s health status and its dynamics.

Spousal bereavement, i.e. death of a spouse, is a major stressor of health, and according to numerous scientific findings appears to be associated with increased hospitalization and mortality hazard [23–31]. The phenomenon in which bereaved individuals have higher mortality risk compared with their non-bereaved counterparts is termed as ‘widowhood effect’[32]. In addition to that, the effect of bereavement on mortality does not appear to behave similarly across different age groups and sexes, also being dependent on the time since the realization of the event [29–31, 33–35]. Apart from the factors above, there is further indication that resilience mechanisms to the stressor is not homogeneous across individuals [36–39].

Since spousal bereavement is one of the most stressful and inevitable encounters during one’s life course [40], there is a need for the creation of tools and computational models which quantify resilience at an individual level. Such tools can be then consequently utilized to extract personalized risks of adverse outcomes, i.e. death, after the stressor’s realization [41, 42]. The aforementioned addresses the gap of the existing literature to address who specifically is at high risk after experiencing bereavement. It needs to be determined whether such a DIORs setting can not only provide useful insights on how human resilience changes in response to bereavement, but also provide individualized risks of approaching a tipping point, i.e., death, after being perturbed by the loss of a loved one, given the resilience status before the event happening.

The aim of this paper is to calculate various metric-based indicators, i.e., DIORs, to capture changes in resilience in response to loss of one’s spouse, as well as function as predictors of individualized mortality risks after. To this end, we studied the association of spousal bereavement—as a standardized stressor—with dynamics of health status, as manifested in the healthcare consumption of Danish elderly over the age of 65. The idea is that healthcare consumption can serve as an indicator variable of a person’s health and resilience. We first aim to analyse the behaviour of these indicators, pre- and post- bereavement reflecting loss of resilience. Our second aim is to determine whether these indicators are predictors of failure by testing the hypothesis of them being associated with death, an ultimate ‘tipping point’ upon perturbation. The aforementioned would be fulfilled by observing significantly higher mortality in the groups of people with the highest levels of these indicators before bereavement.

## Methods

### Data processing

Data were collected from national population registers of Statistics Denmark (https://www.dst.dk) containing information on the healthcare consumption of Danish Elderly over the age of 65, who had been resident in Denmark for at least 5 years before the date of death, from January 1^st^, 2011 to December 31^st^, 2016 [43, 44]. Healthcare consumption was divided into weekly measurements and was initially grouped into different kinds of costs, while being measured in thousands of Danish Kroners (1 unit = 1000 DKKR). Information on prescription drug, hospital (inpatient and outpatient), home care, residential care, and primary care costs was available and a new variable, aggregating all the types of healthcare costs, was constructed for each individual, Variables describing sex, date of birth, date of death and bereavement date (date when spouse died) were virtually complete.

### Availability of data & materials

According to Danish law, scientific organizations can be authorized to work with data within Statistics Denmark and can provide access to individual scientists inside and outside of Denmark. Data are available via the Research Service Department at Statistics Denmark: (www.dst.dk/da/TilSalg/Forskningsservice) for researchers who meet the criteria for access to confidential data.

### Definition of DIORs

We computed for each individual the average (AVERAGE) healthcare consumption one year before and after bereavement as a measure of health status. Initial data exploration indicated that many individuals gradually increase their healthcare costs approximately linearly with time. Hence, we decided to compute the slope (SLOPE), i.e. the average linear temporal change of a person’s healthcare costs. Thus, the AVERAGE and SLOPE, serve as indicators which parameterize the overall trend of individuals, being reflected by an average level of expenditures and their respective linear rate of change. We also calculated Dynamical Indicators of Resilience (DIORs) or early warning signals which capture the individuals’ dispersion dynamics from their overall, health expenditures trend. For that purpose, we further computed the mean squared error (MSE) of the linear models regressing health costs on weeks, measuring the magnitude of dispersion and variability around that linear trajectory of costs. Higher values of MSE indicate larger fluctuations from that linear trend of healthcare patterns and may reflect large shifts of health status for the individual, indicative of critical slowing down. Finally, we evaluated the memory of those fluctuations across consecutive weeks, by calculating the lag-1 autocorrelation of the detrended expenditures (residuals) between subsequent moments (lagging by 1 unit of time). High levels of lag-1 autocorrelation are reflective of a stable fluctuating behavior, whereas low ones of a rapid changing one. We note that while there are many summary statistics which describe other aspects of the time-series distribution and are subject to empirical testing, in the current research we restrict to those depicting overall centrality and trend of costs (AVERAGE, SLOPE), as well as those reflecting the magnitude of variability (MSE) and its memory (lag-1 autocorrelation), in similar fashion with previous works on the literature [6, 18, 21, 22], for comparability purposes (See Appendix for the mathematical formulas of DIORs).

### Data analysis

Comparisons of the DIORs one year before and after the spousal bereavement were performed for the whole sample, but also stratified based on sex and various age categories. The distributions of the weekly healthcare costs and the metrics describing them, were positively skewed (50% of the total cost variable had the value 0) and non-normally distributed. Hence, we assessed the significance of the statistical differences in DIORs between the two bereavement periods via non-parametric two-tailed Wilcoxon signed-rank tests.

Comparison analysis of the DIORs one year before bereavement, between individuals who survived within one year after the bereavement event with those who did not, was performed to evaluate the hypothesis of these metrics signaling early loss of health resilience. Statistically significant results were assessed with the use of two-tailed Mann–Whitney U tests.

We deployed quantile-median regression models [45] using each DIOR at a time as response (Y) variable and the age of the individuals as predictor (X), while adjusting for sex, to predict the change in the median of the DIORs in response to an increase in age. Therefore, we evaluated if those dynamic indicators follow the theory of aging, by observing the models’ slope-coefficient, with older individuals becoming frailer, thus exhibiting higher DIORs values. Ιt should be noted that the slopes of the median regression models are different from the SLOPE DIOR, with the former indicating the between-individuals variability and the latter the within-individual one. Sex-based statistical comparisons (two-tailed Mann–Whitney U tests), both pre- and post-bereavement, were furtherly performed to examine if loss of health resilience, thus frailty, is more present in one of the sexes.

To investigate if extreme DIORs of healthcare expenditures before bereavement are predictive of death after the stressor, we performed a time to event (mortality) analysis using Kaplan–Meier survival estimates and Cox regression stratified on sex, with a follow-up period up to one year after the bereavement with the outcome being death from any cause. Each DIOR was categorized into tertiles, with the reference category being the bottom tertile for each respective DIOR. Autocorrelation for those individuals with constantly zero healthcare costs across the follow-up could not be computed and were classified in the bottom tertile of the respective metric as this corresponds to our intuitive expectation that persons with zero healthcare costs are more resilient than persons with varying healthcare costs. We note that some individuals may have healthcare trajectories with many consecutive zeros (i.e., zero healthcare expenses for and extended time-period) leading to high autocorrelation measures, and that for these cases lag-1 autocorrelation is not an appropriate indicator of critical slowing down, as the observed “memory” in the data is not due to a slowing down per se, but rather due to lack of signal. This issue may lead to some noise in the intended use of the lag-1 autocorrelation measure (the intention being that high autocorrelation corresponds to slowing down phenomena indicating higher frailty), and we anticipate that this may cause decrease in the significance of statistical associations between the lag-1 autocorrelation and dependent variables.

To assess the added value of DIORs in the discrimination ability of a Cox regression model including only age to predict one-year mortality after spousal bereavement, we calculated the time-dependent area under the one-year receiver-operating-characteristic curve (AUC) [46–48]. Estimation of prediction error was performed using the leave-one-out-bootstrap method, on 50 bootstrap (drawn with replacement) samples [49]. Within a 1-year timespan, the AUC is the probability of an individual receiving a higher mortality risk, given that he/she died within that year, compared to an individual who survived in the same period. All the analyses were conducted using R version 4.0.3 [50] (See Appendix for the mathematical formulas of the models).

## Results

### Population statistics

**Fig. 1:**
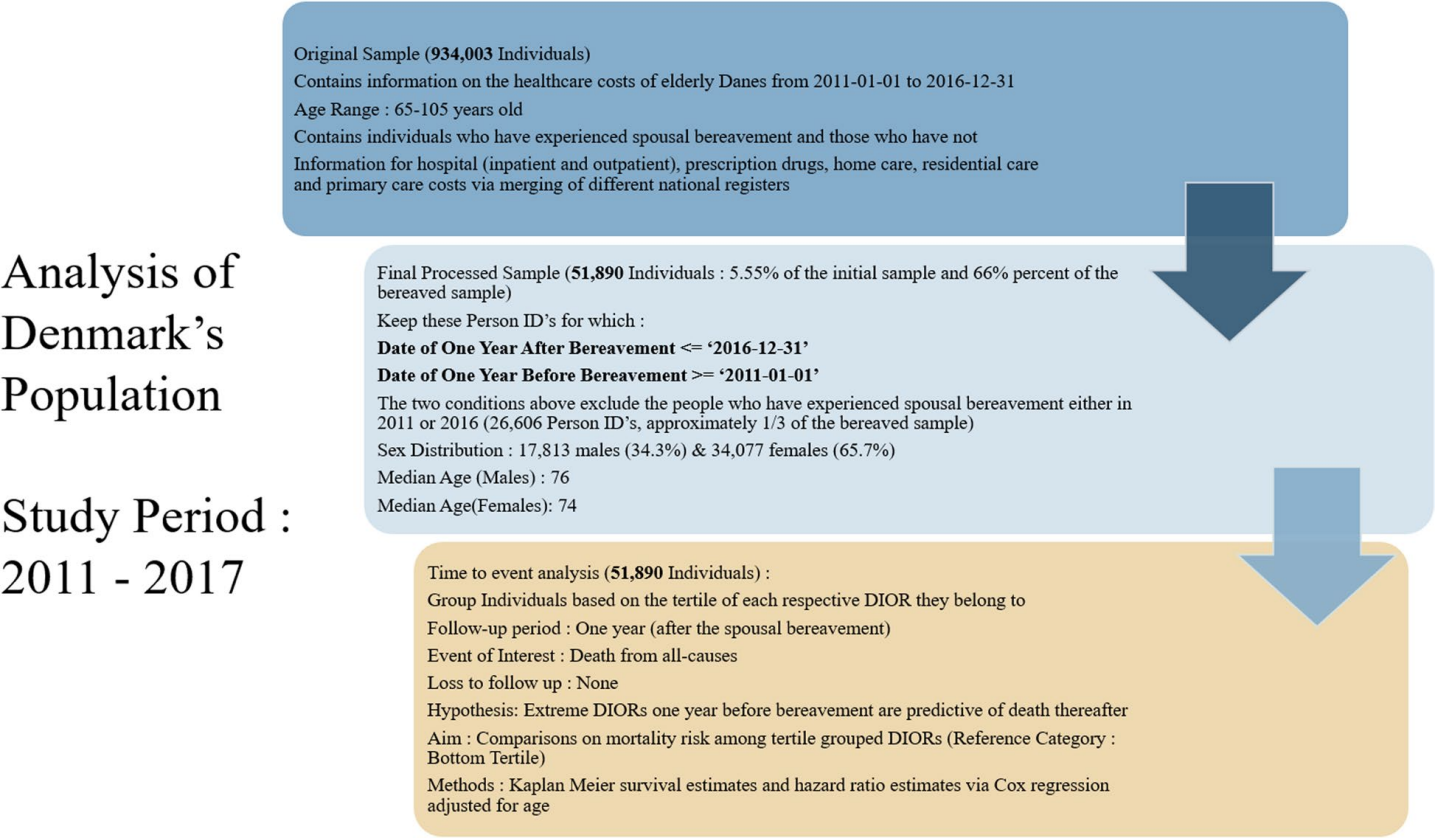
Flowchart of the analysis of Danish healthcare register data. Upper box presents the initial sample of Danes over 65 years for the general population. Middle box explains the process from the original sample towards the final one containing only the spousal bereaved individuals suitable for the analysis. The box at the bottom illustrates the process of time to event analysis

The general population consisted of 934,003 Danish citizens, with their age ranging from 65 to 105 years old. Weekly healthcare expenditures for each individual were available from January 1^st^, 2011, to December 31^st^, 2016. For the purposes of calculating their respective DIORs one year before and after bereavement, our analysis was restricted to 51,890 individuals who experienced a spousal bereavement within the 2012–2015 time period (5.55% of the initial sample). The final sample consisted of 17,813 males (34.3%) and 34,077 females (65.7%) with their median age (interquartile range) at start of the study being 76 (11) and 74 (10) years respectively. From the sample of 51,890 bereaved individuals, 2862 died in the first year thereafter, with more than 90% being followed for one year after bereavement. An illustration of the sample processing is depicted in Fig. 1.

**Fig. 2:**
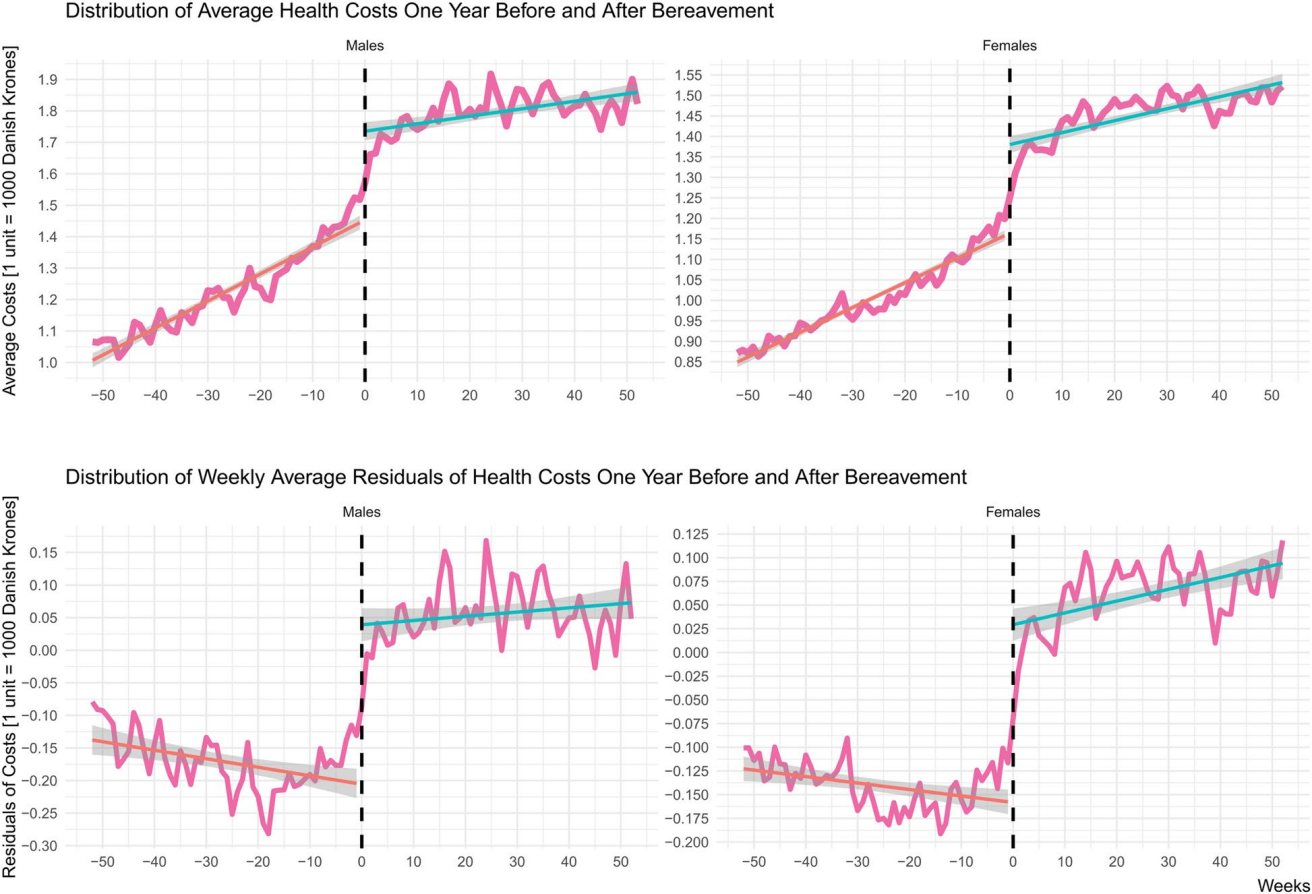
Sex stratified weekly average and residuals of healthcare expenditures pre- and post-bereavement. Week 0 depicts the date of the standardized stressor of bereavement, splitting the time interval one year before and after bereavement. Linear regression lines are fitted both before and after the stressor, indicating the differences in the healthcare consumption pattern between the two periods. Average healthcare consumption after bereavement is higher than before, for both males and females. The average of weekly-based residuals also seems to be following the same pattern, that being increased after bereavement. Females seem to exhibit a more resilient behavior, with smaller deviations when compared with males, manifested in the weekly average and the residuals of their respective healthcare costs

The population average healthcare costs were investigated by a stratified-on sex analysis for the weekly average expenditures and residual time-series and portrayed in Fig. 2. Increasing trends are observed on the weekly average, manifesting the effect of aging in healthcare costs. Both the weekly average costs and residuals are increased after bereavement, a pattern that can be also observed in the sex and age stratified analysis (see Additional File 1; Supplementary Fig. 1 & 2).

### DIORs across bereavement states

A comparison of the dynamical indicators of resilience (DIORs) pre- and post-bereavement is listed in Table 1. The average of healthcare expenditures (DKK * 1000) post-bereavement (median (IQR) = 0.299 (1.377)) was significantly higher (*p*-value < 0.001) than pre-bereavement (median (IQR) = 0.208 (0.697)). A comparison of slope and MSE of healthcare costs pre- and post-bereavement followed a similar pattern. Lag-1 autocorrelation of linearly detrended healthcare costs exhibited a different behaviour than the rest of the metrics, that being not significantly different (*p*-value = 0.065) after bereavement (median (IQR) = 0.019 (0.378)) when compared with before (median (IQR) = 0.017 (0.391).

**Table 1:** Median (IQR) of DIORs one year before and after spousal bereavement in subgroups

Group	DIORs	Pre Bereavement	Post Bereavement	P-value of difference in pairs
All (*N* = 51,890)	Average	0.208 (0.697)	0.299 (1.377)	< 0.001
	Slope	0.007 (7)	0.200 (10)	< 0.001
	Mean Squared Error	0.522 (4.237)	0.715 (6.864)	< 0.001
	Autocorrelation	0.017 (0.391)	0.019 (0.378)	0.065
Males (*N* = 17,813)	Average	0.234 (0.875)	0.355 (1.901)	< 0.001
	Slope	0.020 (8)	0.100 (12)	< 0.001
	Mean Squared Error	0.714 (5.750)	1.020 (9.887)	< 0.001
	Autocorrelation	0.039 (0.446)	0.041 (0.417)	0.011
Females (*N* = 34,077)	Average	0.198 (0.618)	0.277 (1.165)	< 0.001
	Slope	0.000 (6)	0.200 (9)	< 0.001
	Mean Squared Error	0.446 (3.539)	0.592 (5.583)	< 0.001
	Autocorrelation	0.007 (0.358)	0.009 (0.353)	0.613
65–69 (*N* = 12,946)	Average	0.130 (0.355)	0.168 (0.458)	< 0.001
	Slope	0.000 (4)	0.001 (6)	< 0.001
	Mean Squared Error	0.239 (1.541)	0.297 (2.290)	< 0.001
	Autocorrelation	-0.020 (0.225)	-0.020 (0.226)	0.535
70–74 (*N* = 13,361)	Average	0.170 (0.464)	0.229 (0.700)	< 0.001
	Slope	0.000 (4)	0.090 (7)	< 0.001
	Mean Squared Error	0.371 (2.685)	0.485 (3.998)	< 0.001
	Autocorrelation	-0.011 (0.279)	0.009 (0.282)	0.996
75–79 (*N* = 12,046)	Average	0.232 (0.719)	0.339 (1.454)	< 0.001
	Slope	0.020 (7)	0.200 (11)	< 0.001
	Mean Squared Error	0.623 (4.846)	0.890 (8.292)	< 0.001
	Autocorrelation	0.022 (0.387)	0.026 (0.384)	0.775
80–84 (*N* = 8,762)	Average	0.328 (1.372)	0.608 (3.454)	< 0.001
	Slope	0.200 (11)	0.400 (20)	0.015
	Mean Squared Error	1.121 (7.534)	1.752 (13.195)	< 0.001
	Autocorrelation	0.107 (0.505)	0.112 (0.489)	0.444
85plus (*N* = 4,775)	Average	0.736 (3.477)	2.139 (6.952)	< 0.001
	Slope	1.000 (26)	1.100 (39)	0.507
	Mean Squared Error	2.442 (11.412)	3.822 (18.413)	< 0.001
	Autocorrelation	0.316 (0.676)	0.263 (0.572)	< 0.001

*IQR* = Interquartile Range

Slope medians and IQRs have been multiplied by 1000 for digit uniformity across the table

Statistical comparisons indicated that males manifest significantly higher (*p*-value < 0.001) DIORs than females (*p*-value < 0.001), both one year before and after bereavement (Additional File 1; Supplementary Table S1).

We applied quantile-median regression to verify the association between DIORs and aging. When the DIORs were regressed on age, all the extracted slope coefficients were positive and statistically significant (p-value < 0.001) (Additional File 1; Supplementary Table S2). We observed similar trends on the DIORs in both males and females, as well as within the strata of age (see Table 1).

### Predictive value of DIORs before bereavement for death thereafter

In order to assess the hypothesis of DIORs before bereavement being quantitative indicators of the level of resilience of a system, we compared them across those bereaved individuals who survived during the first year after bereavement, i.e. ‘Survivors’ and those who did not, i.e. ‘Deceased’. The average was significantly higher (*p*-value < 0.001) for the latter group (median (IQR) = 1.825 (5.326)) compared with the former (median (IQR) = 0.191 (0.588)). The slope coefficient for the Deceased group (median (IQR) = 0.004 (0.062)) was also significantly higher (*p*-value < 0.001) than the Survivors group (median (IQR) = 0 (0.006)). The MSE exhibited a similar pattern, that being significantly higher (*p*-value < 0.001) for the Deceased (median (IQR) = 8.005 (31.389)) when compared with the Survivors (median (IQR) = 0.456 (3.434)). Lag-1 autocorrelation of detrended healthcare expenses was also found to be significantly higher (*p*-value < 0.001) for those who died earlier (median (IQR) = 0.356 (0.600)) compared with those who survived (median (IQR) = 0.008 (0.360)). Similar differences were found for both sexes separately, as well as in all age restricted strata (statistical-test results available in Additional File 1; Supplementary Table S3, S4 and S5).

**Fig. 3:**
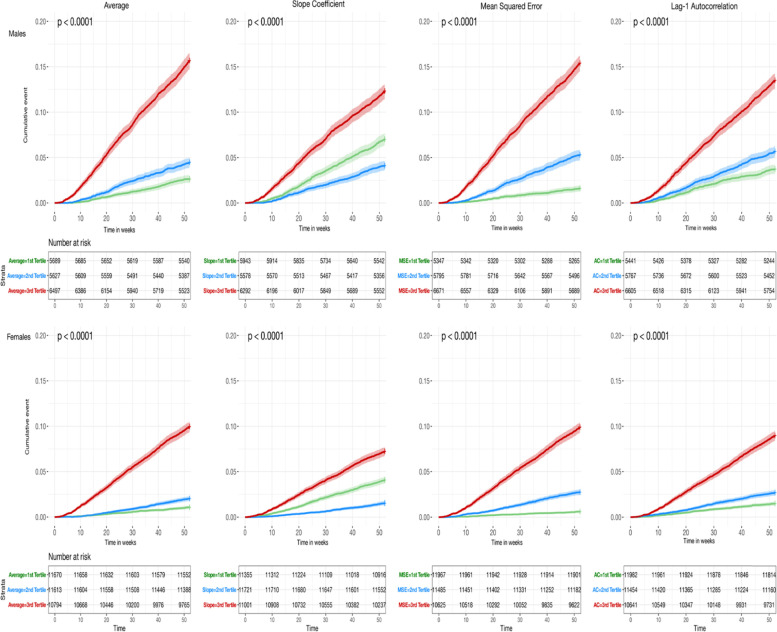
Sex stratified Kaplan–Meier curves of DIORs on cumulative deaths (%) up to one-year after bereavement. Time is measured in weeks with each respective number at risk in the tables below each curve. *P*-values indicate the significance of log-rank test for difference in survival between the tertiles. Shaded areas represent 95% CIs. First row depicts the results for males, whereas second row is for females. The colors depict the tertiles of each respective DIOR (red: top-3^rd^ tertile, blue: middle -2^nd^ tertile, green: bottom-1^st^ tertile)

Time to event analysis was performed to investigate the value of tertile-grouped DIORs before bereavement to predict death in the first year thereafter. The Kaplan–Meier curves of the analysis are presented in Fig. 3, depicting the cumulative percentage of deaths belonging in each tertile group of all the DIORs for the period of 52 weeks after bereavement. The cumulative percentage of deaths within one year after spousal bereavement was the highest for the group of people belonging in the top tertile of each DIOR. The pattern was observed both in males and females, with the latter exhibiting smaller percentage of deaths than the former. The middle and bottom tertile seem to have moderate to weak differences in the survival curves.

The estimated hazard ratios (HRs) of DIORs on mortality risk, along with their respective 95% confidence intervals (CIs), of the Cox regression adjusted for age, are reported in Table 2. When compared with the reference category (bottom tertile), the group of males in the top tertile of average appeared to have a 4.81-fold higher mortality risk [95% confidence interval [CI], 4.04–5.72]. The corresponding mortality risk was found to be 7.28-fold higher [95% CI, 6.03–8.78] for females. Similar risk differences were found for the slope coefficient and MSE. Although we did not necessarily expect autocorrelation to exhibit the same predictive power, a similar trend was observed in the top tertile group for lag-1 autocorrelation. Males exhibited a 2.86-fold higher mortality risk [95% CI, 2.44–3.34] while females’ risk being 4.65-fold higher [95% CI, 3.95–5.48], when compared with the bottom tertile. We further assessed the joint effect of extreme DIORs in mortality risk by creating a Score variable, counting the number of DIORs for which an individual belongs in the top tertile category. The 9% of individuals belonging in the top tertile category of all the DIORs had 14.59-fold [95% CI, 12.33–17.26] higher risk compared with those not belonging into any top tertile DIORs category (Additional File 1; Supplementary Table S6).

**Table 2:** Hazard ratios (95% CI) of all-cause mortality after bereavement dependent on various DIORs in tertiles

Stratum	DIORs	Tertile	HR [95% CI]	*P*-value
All (*N* = 51,890)	Average	Bottom	1	
		Middle	1.64 [1.41, 1.90]	< 0.001
		Top	5.95 [5.24, 6.76]	< 0.001
	Slope	Bottom	1	
		Middle	0.51 [0.45, 0.57]	< 0.001
		Top	1.60 [1.47, 1.74]	< 0.001
	Mean Squared Error	Bottom	1	
		Middle	3.38 [2.84, 4.03]	< 0.001
		Top	10.05 [8.54, 11.84]	< 0.001
	Autocorrelation	Bottom	1	
		Middle	1.59 [1.40, 1.81]	< 0.001
		Top	3.66 [3.27, 4.10]	< 0.001
Males (*N* = 17,813)	Average	Bottom	1	
		Middle	1.57 [1.28, 1.92]	< 0.001
		Top	4.81 [4.04, 5.72]	< 0.001
	Slope	Bottom	1	
		Middle	0.64 [0.55, 0.76]	< 0.001
		Top	1.61 [1.43, 1.81]	< 0.001
	Mean Squared Error	Bottom	1	
		Middle	2.83 [2.23, 3.60]	< 0.001
		Top	7.73 [6.19, 9.65]	< 0.001
	Autocorrelation	Bottom	1	
		Middle	1.44 [1.21, 1.71]	< 0.001
		Top	2.86 [2.44, 3.34]	< 0.001
Females (N = 34,077)	Average	Bottom	1	
		Middle	1.74 [1.40, 2.16]	< 0.001
		Top	7.28 [6.03, 8.78]	< 0.001
	Slope	Bottom	1	
		Middle	0.41 [0.34, 0.49]	< 0.001
		Top	1.58 [1.41, 1.78]	< 0.001
	Mean Squared Error	Bottom	1	
		Middle	4.00 [3.10, 5.17]	< 0.001
		Top	12.89 [10.14, 16.40]	< 0.001
	Autocorrelation	Bottom	1	
		Middle	1.75 [1.46, 2.11]	< 0.001
		Top	4.65 [3.95, 5.48]	< 0.001

All hazard ratios (HRs) adjusted for age, stratified on sex, *CI* Confidence Interval

### Discrimination ability of DIORs

We assessed the added value of DIORs to the discrimination ability, next to age, in predicting mortality within one year after spousal bereavement among all the individuals (N = 51,890) aged 65 years and older. Table 3 summarises the results of the AUC analysis stratified on sex. Age seems to be the main factor of discriminative value with an AUC of 68.9% [95% CI, 67.4%—70.3%] and 70.2% [95% CI, 68.8%—71.6%] for males and females, respectively. The addition of average to the model with age, increased AUC by 7.1% [95% CI, 6%—8.3%] and 8.9% [95% CI, 7.7%—10.1%] for males and females, respectively. The discriminative value, based on the AUC, was increased with the inclusion of slope by an extra 2.5% [95% CI, 1.8%—3.2%] for males and by 4.1% [95% CI, 3.2%—4.9%]. The MSE of healthcare consumption improved the discriminatory power (AUC) to predict all-cause mortality by an extra 8.1% [95% CI, 6.9%—9.2%] for males and 10% [95% CI, 8.8%—11.2%] for females. Lag-1 autocorrelation increased the discriminatory value of the model with age, by a further 3.4% [95% CI, 2.6% – 4.3%] in males and 6.5% [95% CI, 5.4% – 7.5%] in females.

**Table 3:** Discrimination ability to predict one-year mortality based on DIORs stratified by sex

	Males (*N* = 17,813, Deceased = 1423)		Females (*N* = 34,077, Deceased = 1439)	
	AUC [95% CI]	*p*-Value	AUC [95% CI]	*p*-Value
Age	68.9 [67.4, 70.3]	< 0.001	70.2 [68.8, 71.6]	< 0.001
Age + Average	76 [74.8, 77.2]	< 0.001	79.1 [78, 80.2]	< 0.001
Age + Slope	71.4 [70.0, 72.7]	< 0.001	74.3 [73.0, 75.5]	< 0.001
Age + MSE	76.9 [75.8, 78.1]	< 0.001	80.2 [79.2, 81.1]	< 0.001
Age + Autocorrelation	72.3 [70.9, 73.6]	< 0.001	76.6 [75.5, 77.8]	< 0.001
Age + Average + MSE + Autocorrelation + Slope	78.5 [77.4, 79.6]	< 0.001	82.4 [81.5, 83.3]	< 0.001

AUC: Area Under the ROC Curve, *P*-values < 0.05 indicate significant difference in AUC compared with the null model (no predictors)

The final model’s predictor variables consisted of age, average, slope coefficient, MSE and lag-1 autocorrelation. The discriminatory power according to the AUC was 78.5% [95% CI, 77.4%—79.6%] for males and 82.4% [95% CI, 81.5%—83.3%] for females. In regards to the model’s covariates, the average indicator was highly correlated with MSE (*ρ* = 0.79, Spearman’s *p*-value < 0.001), while the cross-correlations among the rest of the indicators were ranging from 0.07 to 0.33.

## Discussion

The current research focused on the perturbation of spousal bereavement and the ability of DIORs to capture transitions in human health in response to this perturbation. To this end, we analyzed the healthcare consumption of Danish citizens over the age of 65 by applying the logic of systems failure, as expressed in the dynamical indicators of resilience. Aggregation of all the different types of costs for each person merges all the discrete layers of healthcare together (from treatment to care), thus serving as a read out of their health status and level of resilience.

The analysis of bereaved individuals produced the following observations. First, all the indicators describing the healthcare expenditures data were significantly increasing with age and higher in males, suggesting lower resilience. In addition, the average, slope coefficient and MSE were significantly higher the year after bereavement when compared with the year before. The aforementioned is a strong indication that spousal bereavement seems to be a life-event which apart from causing intense physical and mental distress to peoples’ lives, poses the risk of increased, more volatile healthcare consumption and a rate of increase across time. Last, all the dynamical indicators were found to be signals of early loss of resilience before critical transitions in human health, i.e. -death. We inferred the latter by observing that the group of individuals with the highest indicators of resilience, before bereavement, exhibited a significantly higher mortality risk after bereavement when compared to those with low DIORs. Ultimately, we found evidence that these indicators are able to capture the dynamics of human resilience and predict mortality, indicating that with ageing comes frailty, with males being the frail sex.

Time to event analysis evaluated the hypothesis of DIORs predictive value. Individuals with high DIORs one year before bereavement were found to have significantly increased mortality risk for the year thereafter when compared with low DIORs group. These outcomes were consistent for each respective indicator, both for males and females. Therefore, this finding further enhances the hypothesis of these metrics being indicators of resilience in the life sciences, with higher DIORs indicating less resilient systems.

The AUC analysis indicated that all of the DIORs seem to increase the AUC when included in a model with age. We observed the highest increase in the AUC by adding the MSE, followed by the average, then lag-1 autocorrelation and last, the slope. Since our goal was to evaluate the predictive performance of DIORs on mortality, we implemented both the average and MSE in the final model, even though their correlation was high, following the recommendations of recently published work [51]. The final predictive model with average, MSE, lag-1 autocorrelation, slope, and age as predictors reached an AUC of 78.5% and 82.4% for males and females, respectively. Therefore, it appears that the various signals extracted from the dynamic behavior of health-related time-series are capable of discriminating mortality risks amongst older persons well, both for males and females. The AUC extracted from models of previous studies investigating the added value of various health indicators to mortality predictions in older adults ranged from 0.71 to 0.82 [48, 52]. Thereby, from the abundance of predictive models using various clinical and physiological hallmarks, ours using four dynamical indicators reached a score amongst the highest performing ones, adding increased value in the literature related to prediction of mortality risk at old age.

Analysis of time series data consisting of longitudinal measurements provides the opportunity to study the trajectory of an individual’s health across time, which can be reflected in several dynamical indicators, i.e., variability, rate of change and correlation, moving beyond the mean or just a snapshot value at a given time point. Therefore, we propose a methodological approach that utilizes not only static but also dynamical factors of human health, emphasizing the need for fine-grained, longitudinal measurements in datasets of epidemiological interest. That approach allows for the acquisition of a various, distinct signals which can be further researched for their respective effect, either individually or combined, on specific clinical endpoints. We showed that implementing indicators (average, slope coefficient, MSE, and lag-1 autocorrelation) as covariates which measure dynamical properties of the human health trajectory can increase the predictive performance of models on crucial clinical endpoints, as shown in the values of the hazard ratios and the AUC of the analysis. Therefore, we propose the use of such method on future epidemiological studies of data consisting of longitudinal measurements of health-related variables.

We consider the current study as an extension of the ideas of previous work on complex systems and DIORs, in the field of life sciences [6, 18, 21, 22], aiming to make the logic applicable in register-based longitudinal healthcare data. For that purpose, indicators were chosen primarily based on their ability to capture distinct dynamics of healthcare consumption, without being restricted to match the paradigm of critical slowing down. Interestingly, using DIORs as explanatory variables, along with age and sex, reached an AUC of over 80% for a multifactorial event such as death. We believe that by combining dynamical metrics with more static variables, such as socioeconomic status, education, marital status, Body Mass Index, or comorbidities could greatly enhance the overall performance while also contributing to an even better understanding of resilience and thus death. In addition, the recent advances of wearable and sensor data can provide us with time series of plenty of data, which when analyzed properly by extracting dynamical indicators from them, can depict the dynamics of human health across time. Of course, the applications of these DIORs and their research can be extended beyond healthcare costs, investigating physiological responses across time, such as blood pressure, temperature, glycose levels, electric activity of the heart etc.

The strengths of this research are the sample size, the completeness of healthcare costs, which is based on national register data and is representative of the bereaved population, as well as the time to event analysis to study the behavior of these dynamical indicators in the field of Health sciences. To the best of our knowledge, no other studies under the DIORs framework have used health-expense related variables to quantify human resilience and predict mortality risk after the appearance of a stressor.

However, the study also has its limitations. Our current research uses healthcare expenditures as a measure of health. While in general, the expenditures reflect the health status of individuals and their need for cure and care, there might be potential bias since the null-costs behavior might be explained by the unwillingness of individuals to utilize the medical services rather than their good state of health. Nevertheless, the tax-based healthcare system of Denmark might be one of the factors attenuating the specific bias since it restricts possible financial burden and out of pocket expenses. With regards to the metrics’ computation, while there were over 50 thousand individuals included in the study, in order to study an abrupt stressor such as bereavement, DIORs were measured for a maximum of 52 consecutive weeks before and after bereavement. We are aware that the aforementioned is marginally enough to study the behavior of these indicators and their accompanied dynamics in rolling windows. Regarding the analysis, the results cannot be generalized for the whole population of Denmark since the data include only individuals with age 65 or older who have experienced spousal bereavement. Last, further research is needed to investigate if the constructed tertiles and overall analysis can be applied to bereaved individuals over the age of 65 in countries other than Denmark. The reason for that is that healthcare expenditures are bonded with the healthcare system and social welfare of each country, a factor which may lead to different patterns in the data.

In conclusion, the current research investigated the aggregated time series of healthcare expenditures in Danish bereaved older adults available from the nationwide registers. The tax-based Danish healthcare system, which manages to provide free access to services in its residents, renders those time-series of expenditures proximal data on peoples’ level of health and its dynamics due to the presence of longitudinal measurements. We proposed a novel methodological framework, which via several indicators, investigated the signals of such valuable data and their potential in quantifying health resilience. With regards to the DIORs, we focused on the metrics of average, slope, MSE and lag-1 autocorrelation and evaluated their ability to signal loss of resilience within the health sciences. Spousal bereavement is a stressor which is known to shift the dynamics of human health. Older people who suffer bereavement seem to have increased average consumption, rate of increase, and variability in the healthcare consumption patterns. We showed that these indicators which signal loss of stability in the physical sciences, seem to also be strong predictors of loss of health resilience as well. Indeed, individuals exhibiting high values of the respective DIORs before bereavement, were the ones who had increased mortality risk after the stressor. Hence, the higher these indicators are, the less resilient an individual seems to be. We believe that the implementation of a dynamical approach, which not only considers static predictors but also these ones that capture physiological and dynamic responses (variability, drift), can add significant value to the understanding and assessment of human’s health status.

## Supplementary Information

**Additional file1: Supplementary Figure 1. **Weekly average of healthcare expenditures pre- and post-bereavement between groups. **Supplementary Figure 2.** Weekly average residuals of healthcare expenditures pre- and post-bereavement between groups. **Supplementary Table S1.** Median (IQR) of DIORs between males and females across bereavement periods. **Supplementary Table S2.** Quantile-median regression coefficients of DIORs regressed on age adjusted for sex. **Supplementary Table S3.** Median (IQR) of DIORs between ‘Deceased’ and ‘Survivors’ one year before bereavement for males. **Supplementary Table S4.** Median (IQR) of DIORs between ‘Deceased’ and ‘Survivors’ one year before bereavement for females. **Supplementary Table S5.** Median (IQR) of DIORs before bereavement between ‘Deceased’ and ‘Survivors’ stratified on age categories. **Supplementary Table S6.** Hazard ratios (95% CI) of all-cause mortality after bereavement dependent on DIORs Score

**Additional file2: **Mathematical Formulas of Dynamical Indicators of Resilience (DIORs) and statistical models 

## Data Availability

According to Danish law, scientific organizations can be authorized to work with data within Statistics Denmark and can provide access to individual scientists inside and outside of Denmark. Data are available via the Research Service Department at Statistics Denmark: (www.dst.dk/da/TilSalg/Forskningsservice) for researchers who meet the criteria for access to confidential data.
