# Supplementary material for: Dynamical indicators in time series of healthcare expenditures predict mortality risk of older adults following spousal bereavement
Source: BMC Geriatr. 2022 Apr 8;22:301. doi: 10.1186/s12877-022-02992-x (PMC8991510; doi:10.1186/s12877-022-02992-x)
Supplement: Supplementary file 1 — Additional file1: Supplementary Figure 1. Weekly average of healthcare expenditures pre- and post-bereavement between groups. Supplementary Figure 2. Weekly average residuals of healthcare expenditures pre- and post-bereavement between groups. Supplementary Table S1. Median (IQR) of DIORs between males and females across bereavement periods. Supplementary Table S2. Quantile-median regression coefficients of DIORs regressed on age adjusted for sex. Supplementary Table S3. Median (IQR) of DIORs between ‘Deceased’ and ‘Survivors’ one year before bereavement for males. Supplementary Table S4. Median (IQR) of DIORs between ‘Deceased’ and ‘Survivors’ one year before bereavement for females. Supplementary Table S5. Median (IQR) of DIORs before bereavement between ‘Deceased’ and ‘Survivors’ stratified on age categories. Supplementary Table S6. Hazard ratios (95% CI) of all-cause mortality after bereavement dependent on DIORs Score [file 12877_2022_2992_MOESM1_ESM.docx]

**Supplementary Information**

**Supplementary Tables**

- Supplementary Table S1 : Median (IQR) of DIORs between males and females across bereavement periods
- Supplementary Table S2 : Quantile-median regression coefficients of DIORs regressed on age adjusted for sex
- Supplementary Table S3 : Median (IQR) of DIORs between ‘Deceased’ and ‘Survivors’ one year before bereavement for males
- Supplementary Table S4 : Median (IQR) of DIORs between ‘Deceased’ and ‘Survivors’ one year before bereavement for females
- Supplementary Table S5 : Median (IQR) of DIORs before bereavement between ‘Deceased’ and ‘Survivors’ stratified on age categories
- Supplementary Table S6 : Hazard ratios (95% CI) of all-cause mortality after bereavement dependent on DIORs Score

**Supplementary Figures**

- Supplementary Figure 1 : Weekly average of healthcare expenditures pre- and post-bereavement between groups
- Supplementary Figure 2 : Weekly average residuals of healthcare expenditures pre- and post-bereavement between groups.

**Supplementary Table S1: Median (IQR) of DIORs between males and females across bereavement periods**

| Bereavement Period | DIORs | Males  (N = 17813) | Females  (N = 34077) | P-value of difference  in distributions |
| --- | --- | --- | --- | --- |
| Before Bereavement | Average | 0.234 (0.875) | 0.198 (0.618) | < 0.001 |
|  | Slope Coefficient | 0.021 (8.222) | 0.000 (6.458) | < 0.001 |
|  | Mean Squared Error | 0.714 (5.750) | 0.446 (3.539) | < 0.001 |
|  | Autocorrelation | 0.039 (0.446) | 0.007 (0.358) | < 0.001 |
| After Bereavement | Average | 0.355 (1.901) | 0.277 (1.165) | < 0.001 |
|  | Slope Coefficient | 0.159 (12.764) | 0.171 (9.322) | 0.079 |
|  | Mean Squared Error | 1.020 (9.887) | 0.592 (5.583) | < 0.001 |
|  | Autocorrelation | 0.041 (0.417) | 0.009 (0.353) | < 0.001 |
| IQR = Interquartile Range  Slope medians and IQRs have been multiplied by 1000 for digit uniformity across the table | | | | |

**Supplementary Table S2: Quantile-median regression coefficients of DIORs regressed on age**

**adjusted for sex**

| DIORs | Term | Estimate | P-value | Lower Limit of Confidence Interval | Upper Limit of Confidence Interval |
| --- | --- | --- | --- | --- | --- |
| Average Pre-Bereavement | (Intercept) | 0.2364545 | 0.0000000 | 0.2285199 | 0.2443892 |
|  | Beta for Age | **0.0161818** | 0.0000000 | 0.0154103 | 0.0169534 |
| Average Post-Bereavement | (Intercept) | 0.3984000 | 0.0000000 | 0.3848367 | 0.4119633 |
|  | Beta for Age | **0.0342000** | 0.0000000 | 0.0326709 | 0.0357291 |
| Slope Pre-Bereavement | (Intercept) | 0.0001088 | 0.0000000 | 0.0000811 | 0.0001364 |
|  | Beta for Age | **0.0000217** | 0.0000000 | 0.0000189 | 0.0000246 |
| Slope Post-Bereavement | (Intercept) | 0.0002258 | 0.0000000 | 0.0001772 | 0.0002743 |
|  | Beta for Age | **0.0000322** | 0.0000000 | 0.0000267 | 0.0000378 |
| MSE Pre-Bereavement | (Intercept) | 0.7130000 | 0.0000000 | 0.6808802 | 0.7451198 |
|  | Beta for Age | **0.0585000** | 0.0000000 | 0.0556910 | 0.0613090 |
| MSE Post-Bereavement | (Intercept) | 1.041571 | 0.0000000 | 0.9921249 | 1.091018 |
|  | Beta for Age | **0.0902857** | 0.0000000 | 0.0859690 | 0.0946024 |
| AC Pre-Bereavement | (Intercept) | 0.0430000 | 0.0000000 | 0.0384922 | 0.0475078 |
|  | Beta for Age | **0.0090000** | 0.0000000 | 0.0085644 | 0.0094356 |
| AC Post-Bereavement | (Intercept) | 0.0425625 | 0.0000000 | 0.0384468 | 0.0466782 |
|  | Beta for Age | **0.0090625** | 0.0000000 | 0.0086285 | 0.0094965 |
| AC = Autocorrelation  MSE = Mean Squared Error  Median Regression Model :  $Q_{50}\left( DIOR as continuous variable \right)=Intercept+ Age At Start \left( \mathrm{continuous} \right)+Sex (Categorical)$ | | | | | |

**Supplementary Table S3: Median (IQR) of DIORs between ‘Deceased’ and ‘Survivors’ one year before bereavement for males**

| DIORs  Before Bereavement | Deceased  (N = 1423) | Survivors  (N = 16390) | P-value of difference in distributions |
| --- | --- | --- | --- |
| Average | 1.679 (5.084) | 0.207 (0.687) | < 0.001 |
| Slope Coefficient | 0.003 (0.057) | 0.000 (0.007) | < 0.001 |
| Mean Squared Error | 7.902 (30.207) | 0.585 (4.462) | < 0.001 |
| Autocorrelation | 0.349 (0.641) | 0.024 (0.413) | < 0.001 |
| IQR = Interquartile Range | | | |

**Supplementary Table S4 : Median (IQR) of DIORs between ‘Deceased’ and ‘Survivors’ one year before bereavement for females**

| DIORs  Before Bereavement | Deceased  (N = 1439) | Survivors  (N = 32638) | P-value of difference in distributions |
| --- | --- | --- | --- |
| Average | 1.985 (5.617) | 0.184 (0.545) | < 0.001 |
| Slope Coefficient | 0.004 (0.067) | 0.000 (0.331) | < 0.001 |
| Mean Squared Error | 8.059 (32.264) | 0.400 (2.944) | < 0.001 |
| Autocorrelation | 0.360 (0.565) | 0.000 (0.331) | < 0.001 |
| IQR = Interquartile Range | | | |

**Supplementary Table S5: Median (IQR) of DIORs before bereavement between ‘Deceased’ and ‘Survivors’ stratified on age categories**

| Age Category | DIORs | Deceased  (N = 2862) | Survivors  (N = 49028) | P-value of differences in distribution |
| --- | --- | --- | --- | --- |
| 65-69 | Average  Slope Coefficient  Mean Squared Error  Autocorrelation | 1.184 (3.728)  0.003 (0.065)  13.695 (63.194)  0.255 (0.568) | 0.126 (0.337)  0.000 (0.004)  0.221 (1.350)  -0.021 (0.215) | < 0.001  < 0.001  < 0.001  < 0.001 |
| 70-74 | Average  Slope Coefficient  Mean Squared Error  Autocorrelation | 1.535 (3.796)  0.002 (0.057)  10.293 (47.079)  0.280 (0.528) | 0.163 (0.431)  0.000 (0.005)  0.343 (2.280)  -0.014 (0.263) | < 0.001  < 0.001  < 0.001  < 0.001 |
| 75 - 79 | Average  Slope Coefficient  Mean Squared Error  Autocorrelation | 1.684 (4.923)  0.002 (0.060)  9.468 (39.795)  0.292 (0.540) | 0.216 (0.620)  0.000 (0.007)  0.546 (3.982)  0.013 (0.366) | < 0.001  < 0.001  < 0.001  < 0.001 |
| 80 - 84 | Average  Slope Coefficient  Mean Squared Error  Autocorrelation | 1.706 (5.614)  0.004 (0.057)  6.510 (25.524)  0.338 (0.595) | 0.288 (1.122)  0.000 (0.010)  0.916 (0.010)  0.089 (0.488) | < 0.001  < 0.001  < 0.001  < 0.001 |
| 85 + | Average  Slope Coefficient  Mean Squared Error  Autocorrelation | 2.639 (6.288)  0.005 (0.074)  5.657 (19.151)  0.484 (0.575) | 0.551 (2.692)  0.001 (0.021)  1.956 (10.414)  0.270 (0.656) | < 0.001  < 0.001  < 0.001  < 0.001 |
| IQR = Interquartile Range | | | | |

**Supplementary Table S6: Hazard ratios (95% CI) of all-cause mortality after bereavement**

**dependent on DIORs Score**

| Stratum | DIORs Score | HR [95% CI] | P-value |
| --- | --- | --- | --- |
| All  (N =51,890) | 0  1  2  3  4 | 1  2.72 [2.26, 3.26]  7.59 [6.39, 9.00]  11.36 [9.62, 13.40]  14.59 [12.33, 17.26] | < 0.001  < 0.001  < 0.001  < 0.001 |
| Males  (N =17,813) | 0  1  2  3  4 | 1  2.30 [1.78, 2.96]  6.04 [4.77, 7.65]  8.59 [6.84, 10.80]  10.53 [8.33, 13.31] | < 0.001  < 0.001  < 0.001  < 0.001 |
| Females  (N =34,077) | 0  1  2  3  4 | 1  3.02 [2.31, 3.95]  8.91 [6.96, 11.41]  13.95 [10.98, 17.73]  18.98 [14.89, 24.20] | < 0.001  < 0.001  < 0.001  < 0.001 |

All hazard ratios (HRs) adjusted for age, stratified on sex

CI = Confidence Interval

DIORs Score = The number of DIORs for which the individual belongs to the Top-tertile category


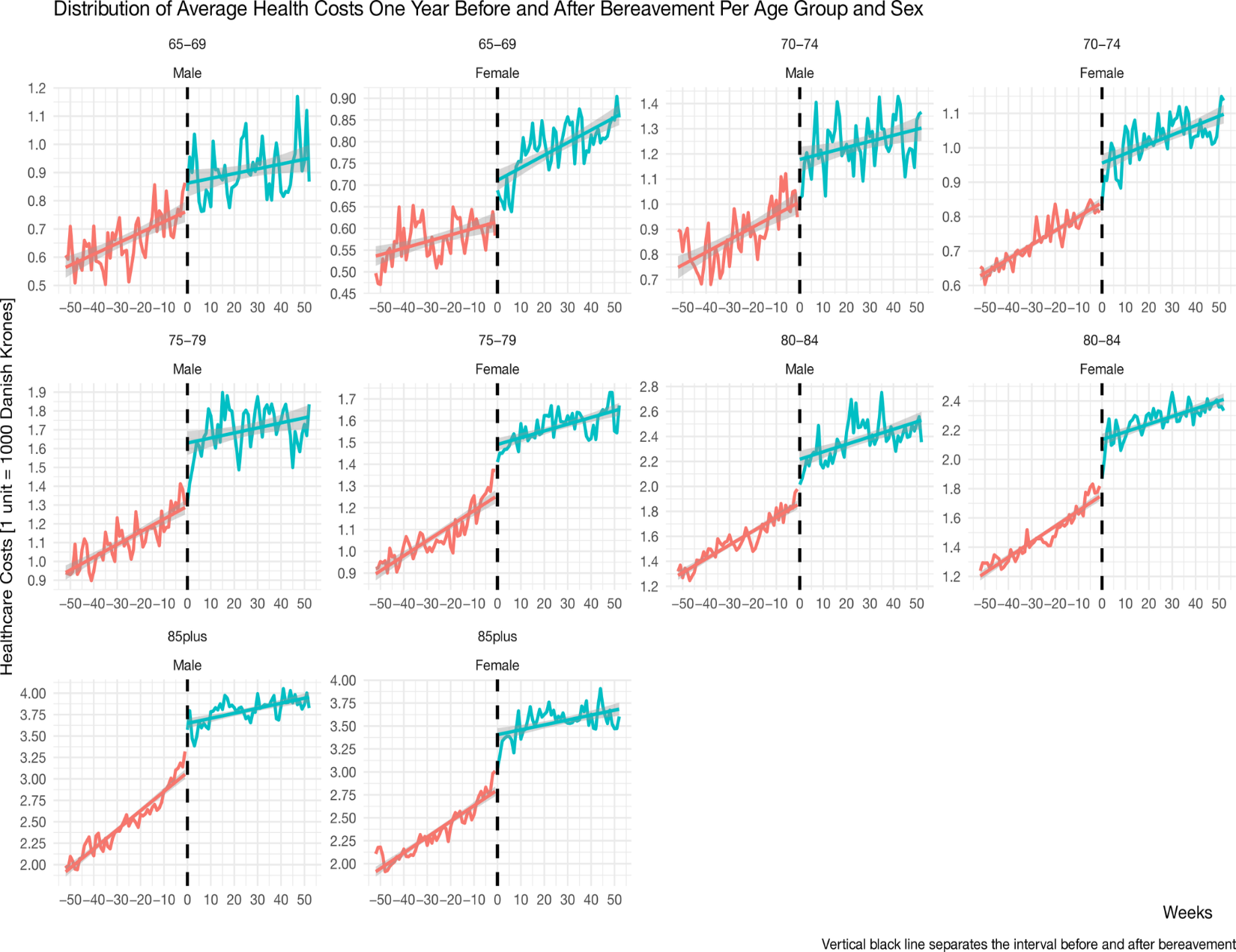


**Supplementary Figure 1: Weekly average of healthcare expenditures pre- and post-bereavement between groups.** Week 0 depicts the date of the standardized stressor of bereavement, splitting the time interval one year before and after bereavement. Linear regression lines are fitted both before (orange) and after (blue) the stressor, indicating the differences in the average of healthcare costs between the two periods. In every age group, average healthcare consumption tends to be increased after spousal bereavement when compared with before. Women spend less on average than men, a pattern that is found on almost all age groups with the exception of the last group (85plus), which appears to behave similarly, for both males and females. As age is increasing, overall average healthcare consumption tends to increase as well.


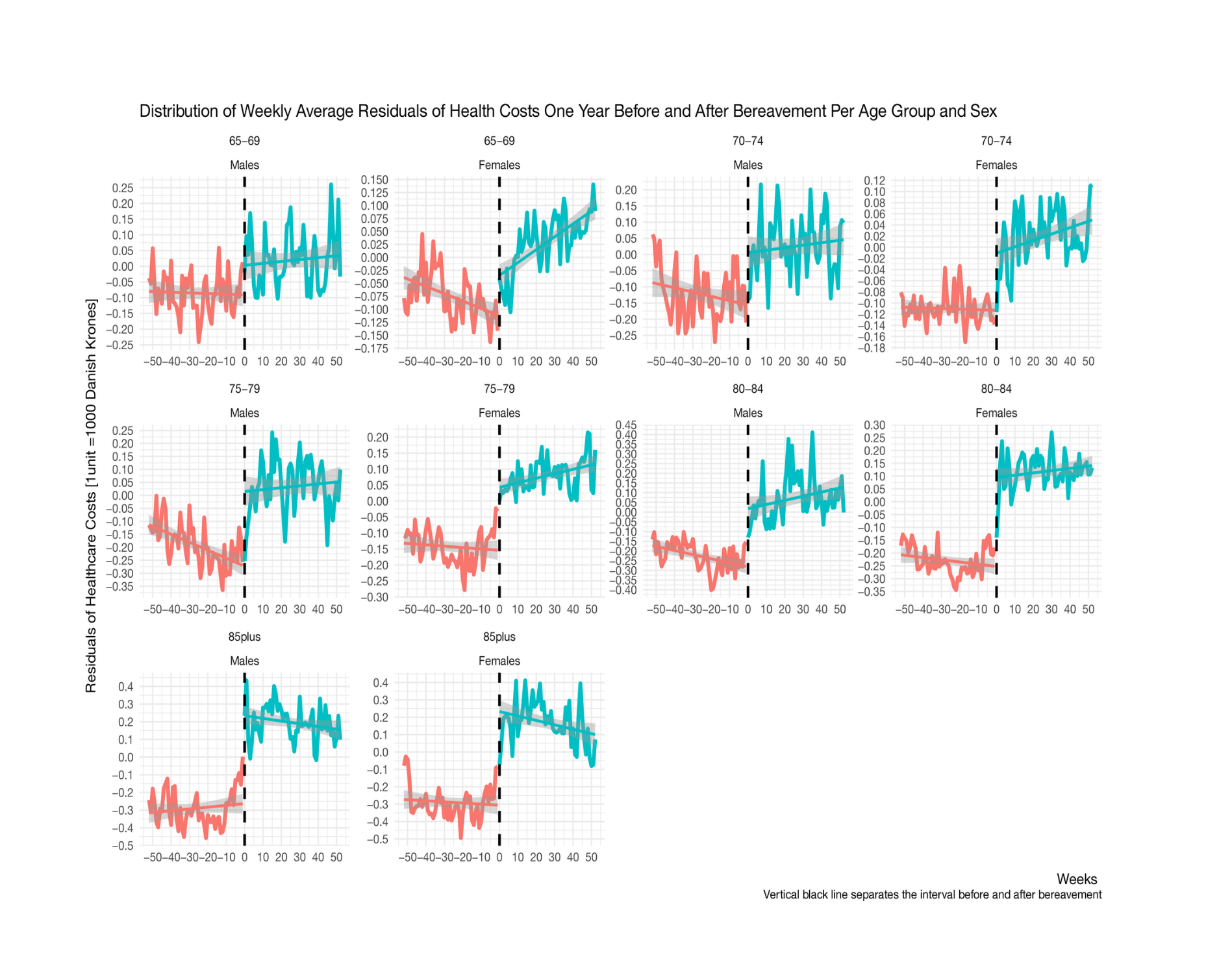


**Supplementary Figure 2: Weekly average residuals of healthcare expenditures pre- and post-bereavement between groups.** Week 0 depicts the date of the standardized stressor of bereavement, splitting the time interval one year before and after bereavement. Linear regression lines are fitted both before (orange) and after (blue) the stressor, indicating the differences in the residuals (observed – predicted values) of healthcare costs between the two periods. The average residuals exhibit a pattern of increase after spousal bereavement, for all age groups and sex. Males seem to exhibit higher deviations compared with females. However, the residuals do not appear to be increasing as age is increasing, a pattern which was found in the weekly average
