# Supplementary material for: Dynamical indicators in time series of healthcare expenditures predict mortality risk of older adults following spousal bereavement
Source: BMC Geriatr. 2022 Apr 8;22:301. doi: 10.1186/s12877-022-02992-x (PMC8991510; doi:10.1186/s12877-022-02992-x)
Supplement: Supplementary file 2 — Additional file2: Mathematical Formulas of Dynamical Indicators of Resilience (DIORs) and statistical models [file 12877_2022_2992_MOESM2_ESM.docx]

Appendix

Mathematical Formulas of Dynamical Indicators of Resilience (DIORs) and statistical models

***Non-model based Indicators:***

- $Average (Mean)=\frac{1}{n}\sum_{i=1}^{n} X_{i}$, where i = 1,2,….,n are the number of weeks and $X_{i}$ is the healthcare expenditures at i-th week.
- $Lag-1 Autocorrelation= \frac{E[\left( y_{t}-\mu\right)\left( y_{t+1}-\mu\right)]}{\sigma_{y}^{2}}$ ,

where μ is the mean and σ is the standard deviation of the variable $y_{t}$ measuring healthcare expenditures at week t .For time series which are linearly detrended, we define $y_{t}$ as the extracted residuals of the linear regression model, discussed below. In similar fashion to the correlation metric, autocorrelation can be either positive or negative. Its range varies from -1 (perfectly negative autocorrelation) to 1 (perfectly positive autocorrelation). Positive autocorrelation means that the increase observed in a time interval (t) leads to a proportionate increase in the lagged time interval (t+1). On the contrary, an increase in an observed time interval (t) leading to a proportionate decrease in the lagged time interval (t+1), is an indication of negative autocorrelation.

***Model-based Indicators :***

Given the used simple linear regression model in the analysis :

$$Basic Regression Model :Y=a+\beta\times X+Error$$

the response variable Y measures the amount of healthcare expenditures, while the X is

called the independent variable, measuring weeks. The model is applied for each individual

separately.

The β value is the **SLOPE coefficient** (the expected increase of Y after a 1-unit increase in X) , whereas α is the intercept of the line (expected value of Y, when X = 0).

1. **The Mean Squared Error (MSE)** of the time series measures the average of the squared deviations (residuals).

$$MSE= \frac{1}{n}* \sum_{i=1}^{n} \left( Y_{i observed}- Y_{i predicted} \right)^{2}$$

where $Y_{i observed}- Y_{i predicted}$ are the residuals of the linear regression model, i.e., the deviations of the predictions of the model from the true observed values of the response variable Y. The MSE is calculated for each individual separately.

- Calculation of DIORs and data handling were performed using the ‘tidyverse’ family of packages in R.
- Statistical models used in the analysis were fitted using the ‘survival’ package in R.
- Predictive performance of fitted models was evaluated using the ‘riskRegression’ package.

**For Table 2 :**

The general Cox-regression model :

Let $x_{1 ,}x_{2},\ldots.,x_{n}$ be the measurements of the n predictor-independent variables $X_{1},X_{2},\ldots,X_{n}$. The Cox regression model estimates the hazard function $h(t)$, which is the probability or the instantaneous risk of dying at time t, as follow :

$$h(t)=h_{0}(t)\times exp(b_{1}\times x_{1}+b_{2}\times x_{2}+...+b_{n}\times x_{n})$$

The purpose of the model is to simultaneously assess the effect of several risk factors (independent variables) on survival, meaning that it allows for the examination of how these risk factors can influence the rate of a particular event of interest happening, such as death or disease infection. In the above formula :

- t can be defined as the survival time
- h(t) can be defined as the hazard function given the set of independent variables (covariates)
- the coefficients ($b_{1 ,}b_{2},\ldots.,b_{n}$) measure the effect size of each respective covariate
- $h_{0}$ is termed as the baseline hazard, corresponding to the hazard when all the covariates are having the value 0.
- For the Average indicator the Cox-model has : $X_{1}$ = Tertiles of Average Consumption for each individual (categorical) and $X_{2}$ = Age at Start of the study (continuous)
- For the Slope indicator the Cox-model has : $X_{1}$ = Tertiles of Slope Coefficient for each individual (categorical) and $X_{2}$ = Age at Start of the study (continuous)
- For the MSE indicator the Cox-model has : $X_{1}$ = Tertiles of Mean Squared Error (MSE) for each individual (categorical) and $X_{2}$ = Age at Start of the study (continuous)
- For the Lag-1 Autocorrelation indicator the Cox-model has : $X_{1}$ = Tertiles of Lag-1 Autocorrelation for each individual (categorical) and $X_{2}$ = Age at Start of the study (continuous)

**For Table 3:**

The mathematical formulas for the models below were fitted exactly as those in Table 2 :

- Age
- Age + Average
- Age + Slope
- Age + MSE
- Age + Lag-1 Autocorrelation

The exact mathematical formula of the model: Age + Average + Slope + MSE + Lag-1 Autocorrelation :

- For males the Cox-model has : $X_{1}$ = Age At start of study (continuous), $X_{2}$ = Tertiles of Average (categorical), $X_{3}$ = Tertiles of Slope Coefficient (categorical), $X_{4}$ = Tertiles of MSE (categorical), $X_{5}$ = Tertiles of Lag-1 Autocorrelation (categorical)
- For females the Cox-model has : $X_{1}$ = Age At start of study (continuous), $X_{2}$ = Tertiles of Average (categorical), $X_{3}$ = Tertiles of Slope Coefficient (categorical), $X_{4}$ = Tertiles of MSE (categorical), $X_{5}$ = Tertiles of Lag-1 Autocorrelation (categorical)

**Coding Example:**

# Load the ‘survival’ package to perform cox regression

library(survival)

# Fit the Cox model with all the predictors

cox_final_model <- coxph(Surv(Time,Event) ~ Average_Tertile + Age_At_Start + Autocorrelation_Tertile + Slope_Tertile + MSE_Tertile, data = data, x = T, y = T)

# Load the ‘riskRegression’ package to evaluate the discriminative performance of the model

library(riskRegression)

# Use the Score function which extracts the performance measures

Cox_Score <- Score(object = list(‘Cox Predictive Model’ = cox_final_model), times = 52,

formula = Surv(Time,Status) ~ 1, se.fit = T, metrics = ‘AUC’, data = data,

split.method = ‘loob’, B = 50)

# We specify times = 52, since we are interested in a prediction time horizon of 52 weeks = 1 year.

# We evaluate the performance using leave-one-out bootstrap using B = 50 bootstrapped samples with replacement.

# We use se.fit = T, to get confidence intervals of our estimates.

# The formula argument specifies the null model which does not make use of any predictors for mortality risk for comparison purposes.

# Get the summary of the score

summary(Cox_Score)

# We can compare two or more different models by specifying them in the Score function in the object argument:

# Let’s create a model which only uses age to predict the event :

cox_only_age <- coxph(Surv(Time,Event) ~ Age_At_Start, data = data, x = T, y = T)

# Now compare

Cox_Compare <- Score(object = list(‘Cox Predictive Model’ = cox_final_model, ‘Cox Only Age’ = cox_only_age), times = 52, formula = Surv(Time,Status) ~ 1, se.fit = T, metrics = ‘AUC’, data = data,

split.method = ‘loob’, B = 50)

summary(Cox_Compare)
